# The Beneficial Effect of Carvacrol in HL-1 Cardiomyocytes Treated with LPS-G: Anti-Inflammatory Pathway Investigations

**DOI:** 10.3390/antiox11020386

**Published:** 2022-02-15

**Authors:** Guya Diletta Marconi, Ylenia Della Rocca, Luigia Fonticoli, Simone Guarnieri, Simone Carradori, Thangavelu Soundara Rajan, Jacopo Pizzicannella, Francesca Diomede

**Affiliations:** 1 Department of Medical, Oral and Biotechnological Sciences, University “G. d’Annunzio” Chieti-Pescara, Via dei Vestini, 31, 66100 Chieti, Italy; guya.marconi@unich.it; 2 Department of Innovative Technologies in Medicine & Dentistry, University “G. d’Annunzio” Chieti-Pescara, Via dei Vestini, 31, 66100 Chieti, Italy; ylenia.dellarocca@unich.it (Y.D.R.); luigia.fonticoli@unich.it (L.F.); francesca.diomede@unich.it (F.D.); 3 Department of Neuroscience, Imaging and Clinical Sciences, Center for Advanced Studies and Technology (CAST), University “G. d’Annunzio” Chieti-Pescara, Via dei Vestini, 31, 66100 Chieti, Italy; simone.guarnieri@unich.it; 4 Department of Pharmacy, University “G. d’Annunzio” Chieti-Pescara, Via dei Vestini 31, 66100 Chieti, Italy; simone.carradori@unich.it; 5 Department of Biotechnology, Karpagam Academy of Higher Education, Coimbatore 641 021, India; drsoundararajan.t@kahedu.edu.in; 6 Ss. Annunziata Hospital, ASL 02 Lanciano-Vasto-Chieti, 66100 Chieti, Italy

**Keywords:** carvacrol, inflammasome, antioxidant, cardioprotective, natural compounds, HL-1 cell line

## Abstract

Carvacrol (CAR), a natural phenolic monoterpene, possesses different biological activities, such as anti-inflammatory and antioxidant activities. The current study aimed to evaluate the response of HL-1 cardiomyocytes to an inflammatory stimulus triggered by lipopolysaccharide from *Porphyromonas gingivalis* (LPS-G), alone or in co-treatment with CAR, to investigate the potential protective role of CAR in the inflammatory process through modulation of the TLR4/NFκB/NALP3/IL-1β pathway and ROS production. In an in vitro experiment, HL-1 cardiomyocytes were exposed to LPS-G and incubated with CAR. We evaluated the anti-inflammatory effect of CAR by the reduction in TLR4, NFκB, NALP3, and IL-1β expression using immunofluorescence staining. Western blot analysis also validated the modulation of the TLR4/NFκB/NALP3/IL-1β pathway. ROS analyses confirmed the protective effects of CAR. Our results suggest that CAR could provide a significant protection role against inflammatory stimulus generated by LPS-G, involving the suppression of the TLR4/NFκB/NALP3/IL-1β signaling pathway.

## 1. Introduction

Carvacrol (CAR) is a natural compound isolated from essential oils, and several studies have reported its antibacterial, antioxidant, anti-inflammatory, fungicidal, insecticidal, and chemopreventive activities [1,2].

Compounds derived from natural plants have attracted a lot of interest in the scientific community because they represent an interesting alternative to synthetic molecules. CAR is a monoterpenoid phenol, identified in high concentrations in essential oils, such as oregano (*Origanum vulgare*), thyme (*Thymus vulgaris*), pepperwort (*Lepidium flavum*), wild bergamot (*Citrus aurantium bergamia*), black cumin (*Nigella sativa*), and fruits of Ajwain (*Carum copticum*) [3,4,5,6,7].

For a long period, CAR has largely been utilized as a food or food additive in the food industry. CAR and thymol, the main constituents of oregano extract essential oil, are responsible for its antioxidant and antibacterial activities [8]. Natural products, such as essential oils and their constituents, have also been shown to be potential and promising drug molecules for the development of therapeutic compounds against cardiovascular diseases [9]. These outcomes have revealed novel and unidentified cardiovascular properties of these natural molecules, opening a new frontier with respect to their common utilization [10]. The cardioprotective role of CAR has already been recognized, with previous papers reporting a decrease in infarction in Wistar rats, and a decrease in cardiac injury markers, such as creatine kinase, creatine kinase-MB, cardiac troponin T, and lactate dehydrogenase, in animals treated with CAR [11,12].

Several studies have already demonstrated that CAR possesses anti-inflammatory properties. In a previous paper, CAR was shown to have the capability to reduce the levels of inflammatory cytokines and the expression levels of inducible nitric oxide synthase (iNOS) and cyclooxygenase (COX)-2 in ischemic cortical tissues [13]. In a paper published by Canbek et al., the role of CAR in preserving the liver against ischemia/reperfusion damage in rats was shown [14]. These findings suggest that CAR could potentially have a cardioprotective effect against myocardial ischemia/reperfusion injury [15]. It is well known that NALP3 inflammasome plays a central role in the development and progression of cardiovascular diseases [16].

Based on the literature, heart failure is one of the complications that may be linked with periodontal disease [17]. *P. gingivalis* is one of the bacteria correlated with periodontal illness and its invasion into the blood results in sepsis and systematic inflammation [18]. Moreover, after *P. gingivalis* invasion, circulating endotoxin is largely linked with the development of heart damage. Lipopolysaccharide from *P. gingivalis* (LPS-G) represents an endotoxin and virulence factor.

Based on this knowledge, the purpose of the current work was to analyze the response of the HL-1 cell line to LPS-G, alone or in co-treatment with CAR, to evaluate the potential protective role of CAR in the inflammatory process through modulation of the TLR4/NFκB/NALP3/IL-1β pathway.

## 2. Materials and Methods

### 2.1. Drugs and Reference Compound

**Figure 1:**
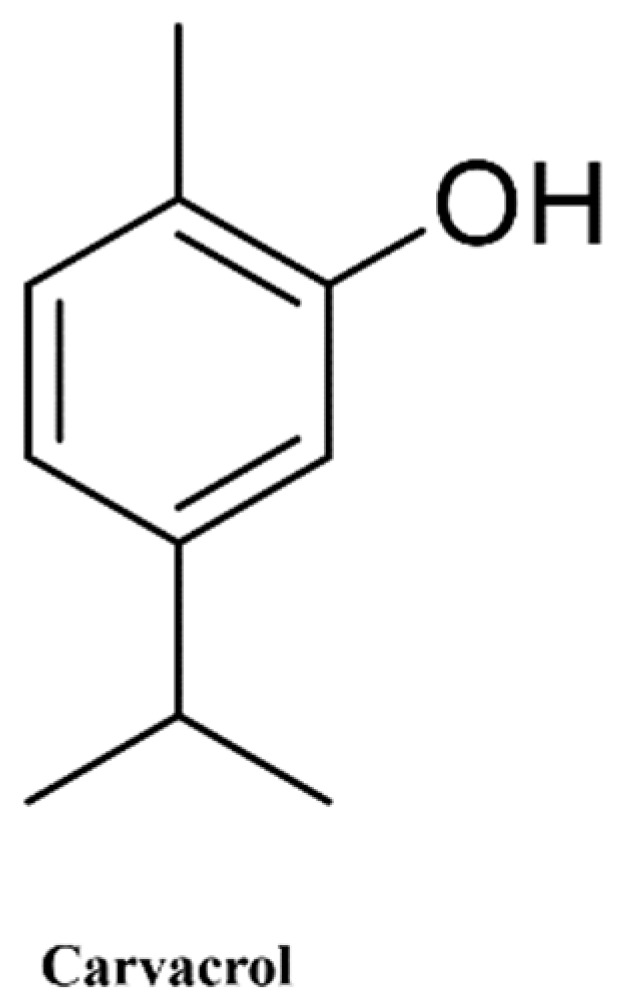
Chemical structure of carvacrol.

Carvacrol (99% purity) was purchased from Sigma-Aldrich (Milan, Italy) (Figure 1).

### 2.2. Cell Culture

HL-1 cells (Sigma-Aldrich, Milan, Italy) were maintained in Claycomb medium completed with 10% fetal bovine serum (Euroclone, Milan, Italy), 2 mM l-glutamine, 0,1 mM norepirephrine, and 100 μg/mL penicillin/streptomycin (Lonza, Basel, Switzerland). The cells were maintained at 37 °C in a humidified atmosphere of 5% of CO_2_ in air and subculture until they reached 80% confluence (Video S1).

### 2.3. Experimental Study Design

All experiments were performed in triplicate with HL-1. The study design is reported as follows:

(i) Untreated HL-1, used as a negative control (CTRL);
(ii) HL-1 treated with 5 μg mL^−1^ of LPS-G for 24 h;
(iii) HL-1 treated with CAR (6.25 μM) for 24 h;
(iv) HL-1 treated with CAR (50 μM) for 24 h;
(v) HL-1 treated with 5 μg mL^−1^ of LPS-G for 24 h and CAR (6.25 μM);
(vi) HL-1 treated with 5 μg mL^−1^ of LPS-G for 24 h and CAR (50 μM).

### 2.4. Cell Viability Assay

The HL-1 cardiomyocyte cells were seeded at a cell density of 6000/well into a 96-well tissue culture plate. The cell metabolic activity of HL-1 cell lines was evaluated after 24, 48, and 72 h of treatment with CAR at 6.25, 12.5, 25, and 50 µM and at 6.25 and 50 µM alone or in co-treatment with LPS (5 µg/mL) on a 96-well polystyrene plate using the 3-(4,5-dimethylthiazol-2-yl)-5-(3-carboxymethoxyphenyl)-2-(4-sulfo-phenyl)-2H-tetrazolium (MTS) assay (CellTiter 96^®^ Aqueous One Solution Cell Proliferation Assay, Promega, Madison, WI, USA). At the established time points, 20 μL per well of MTS dye reagent were placed in the culture medium, and HL-1 cells were placed in an incubator for 3 h at 37 °C [19]. The amount of formazan product was measured at the 490 nM wavelength with a Synergy™ HT Multi-detection microplate reader (Biotech, Winooski, VT, USA), which measured the number of live cells [20]. Data are representative of three independent experiments performed in triplicate.

### 2.5. Confocal Microscopy (CLSM)

The HL-1 cells were seeded at 8500/well on 8-well culture glass slides (Corning, Glendale, Arizona, USA), treated with CAR at 6.25 and 50 µM alone or in co-treatment with LPS-G (5 µg/mL). Then, the samples were fixed for 1 h with 4% paraformaldehyde in 0.1 M of PBS (pH 7.4) (Lonza, Basel, Switzerland) at room temperature. Successively, several washes were performed. Then, the immunofluorescence assays on the specimens were performed as previously described [21]. Successively, the permeabilization of HL-1 was conducted with 0.5% Triton X-100 in PBS buffer (Lonza) for 10 min and stopped with 5% skimmed milk in PBS for 1 h [22]. The primary antibodies used in the study are as follows: anti-TLR4 (1:200) (sc-293072, Santa Cruz Biotechnology, Dallas, TX, USA), anti-NFκB (1:200) (sc-8008, Santa Cruz Biotechnology), anti-NALP3 (1:200) (sc-134306, Santa Cruz Biotechnology), and anti-IL-1β (NB600-633, Novus, Centennial, CO, USA). Then, HL-1 cells were incubated with primary antibodies at room temperature for two hours. Afterward, specimens were incubated with secondary antibody Alexa Fluor 568 red fluorescence-conjugated goat anti-rabbit at a 1:200 dilution (A11031, Molecular Probes, Invitrogen, Eugene, OR, USA) for 1 h at 37 °C. To dye the cytoskeleton actin, HL-1 cells were incubated with Alexa Fluor 488 phalloidin green fluorescent conjugate (1:200) (A12379, Molecular Probes) for 1 h, and to dye the nuclei, HL-1 cells were dyed with TOPRO (1:200) (T3605, Molecular Probes) for 1 h. The Zeiss LSM800 confocal system (Carl Zeiss, Jena, Germany) was utilized to obtain images.

### 2.6. Western Blotting Analysis

The lysates of HL-1 (50 µg) underwent electrophoresis and were moved to a polyvinylidenfluoride (PVDF) membrane. Successively, the membranes were blocked in 5% non-fat milk in PBS 0.1% Tween-20, and then the blotted membranes were incubated overnight at 4 °C with the following primary antibodies: anti-TLR4 (1:500) (sc-293072, Santa Cruz Biotechnology), anti-NFκB (1:500) (sc-8008, Santa Cruz Biotechnology), anti-NALP3 (sc-134306, Santa Cruz, Biotechnology), anti-IL-1β (1:200) (NB600-633, Novus), and β-actin as loading control (1:750, Santa Cruz Biotechnology). After five washings with 0.1% Tween-20 in PBS, the membranes were incubated for 1 h at room temperature with peroxidase-conjugated secondary antibody anti-mouse (A90-116P Goat anti-mouse) and rabbit (A 120-101P Goat anti-rabbit) 1:5000 diluted in 1X PBS, 2.5% milk, and Tween-20 at 0.1% [23]. The levels of expression of the protein were detected using the enhanced chemiluminescence exposure process (ECL) (Amersham Pharmacia Biotech, Milan, Italy) with an image documenter Alliance 2.7 (Uvitec, Cambridge, UK). The detected signals were analyzed by ECL enhancement and assessed through UVIband-1D gel analysis (Uvitec). The data obtained were normalized with values assessed by densitometric analysis of the β-actin protein.

### 2.7. Reactive Oxygen Species (ROS) Evaluation

HL-1 cells were seeded at 85000/well in a 35 mm imaging dish (µ-Dish, ibidi GmbH, Gräfelfing, Germany). Then, HL-1 cells were placed in culture medium containing 6.25 and 50 µM CAR alone or in co-treatment with LPS (5 µg/mL) for 24 h. After 24 h, the medium was replaced with Normal External Solution (NES) containing (in mM) 125 NaCl, 5 KCl, 1 MgSO_4_, 1 KH_2_PO_4_, 5.5 glucose, 1 CaCl_2_, and 20 4-(2-hydroxyethyl)-1-piperazineethanesulfonic acid (HEPES), pH 7.4 and then incubated with 10 μM of 2′,7′-dichlorodihydrofluorescein diacetate (H2DCFDA, Thermo Fisher, Waltham, MA, USA) in a humidified incubator for 30 min at 37 °C, keeping the appropriate culture medium administration for the entire processes. After the incubation with the dye, HL-1 cells were rinsed with NES and detected in NES alone (HL-1) or maintained in NES plus LPS-G, LPS-G, and CAR at 6.25 or 50 µM or CAR alone. Using a confocal microscope, images were casually taken for each experimental condition through a motorized table SMC 2009 and the multiple single position acquisition function (Tiles-Advanced setup, carrier 35 mm petri dish) of Zen Blue software (Zen 3.0 SR, Carl Zeiss) utilizing a Zeiss LSM800 microscope (Carl Zeiss) furnished with an inverted microscope Axio-obserber D1 (Carl Zeiss) and an objective W-Plan-Apo 40 X/1.3 DIC (Carl Zeiss). At 488 nm, the excitation and emission were detected with the filter set over 505–530 nm. During the acquisition of the sample images, the acquisition settings were unchanged. The analyses of the offline pictures were executed utilizing Fiji distribution of ImageJ (version 1.53c, National Institutes of Health, Bethesda, MD, USA) quantifying the mean of fluorescence intensity (arbitrary units, F) and the area of the HL-1 measured (µm^2^) for each obtained cell [24]. Quantitative data of the amount of ROS are expressed as ratio F/µm^2^.

### 2.8. Statistical Analysis

Statistical significance was established with GraphPad 5 (GraphPad, San Diego, CA, USA) software utilizing the *t*-test and ordinary one-way ANOVA and then by post hoc Bonferroni’s multiple comparisons analysis. Values of *p* < 0.05 were considered as statistically significant.

## 3. Results

### 3.1. Effects of CAR Alone or in Co-Treatment with LPS-G on HL-1 Cells’ Metabolic Activity

**Figure 2:**
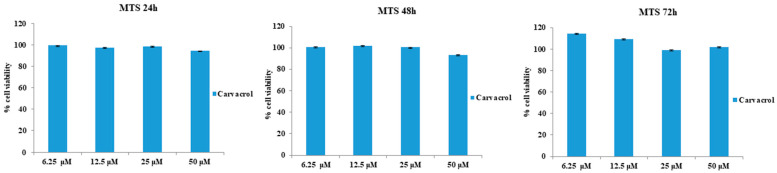
Cell metabolic activity of HL-1 cells treated with 6.25 to 50 μM CAR for 24, 48, and 72 h. Metabolic activity was assessed using the MTS assay and normalized to control cells treated with DMSO (0.2% as the final concentration).

**Figure 3:**
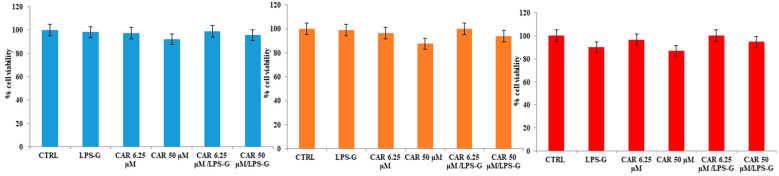
Cell viability of HL-1 cells treated with CAR alone or in co-treatment with LPS-G (5 μg/mL) at different time points of 24, 48, and 72 h. Metabolic activity was assessed using the MTS assay and normalized to control cells treated with DMSO (0.2% as the final concentration).

The effects of CAR alone or in combination with LPS-G on cardiomyocytes of the HL-1 cell line were evaluated using the MTS assay. Initially, CAR was tested individually at concentrations ranging from 6.25 to 50 µM to evaluate the effect on cell viability. CAR appears to be well tolerated in HL-1 cell lines considering that the percentage of viable cells was not affected by CAR treatment at 6.25, 12.5, 25, and 50 µM after 24, 48, and 72 h (Figure 2). Instead, cell metabolic activity was reduced when treated with LPS-G at 5 μg/mL compared to the untreated cells after 72 h of treatment with CAR. Then, CAR was tested at 6.25 and 50 µM alone or in combination with LPS-G (5 μg/mL). After 72 h of treatment, the co-treatment of CAR/LPS-G showed a higher percentage of viable cells compared to the cells treated with LPS-G (5 μg/mL) (Figure 3).

### 3.2. Expression Levels of TLR4/NFκB/NALP3/IL-1β in CAR, LPS-G, and in Combination CAR/LPS-G-Treated Cells

**Figure 4:**
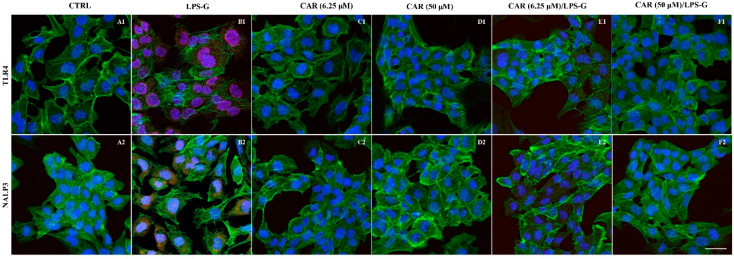
TLR4/NALP3 signaling pathway and differences in the protein levels in HL-1 cell lines. Expression of TLR4 and IL-1β, analyzed by confocal microscopy (**A1**–**F1**), TLR4 expression in untreated cells (CTRL), HL-1 treated with LPS-G, CAR (6.25 μM), CAR (50 μM), CAR (6.25 μM)/LPS-G, CAR (50 μM)/LPS-G. (**A2**–**F2**) NALP3 expression in untreated cells (CTRL), HL-1 treated with LPS-G, CAR (6.25 μM), CAR (50 μM), CAR (6.25 μM)/LPS-G, CAR (50 μM)/LPS-G. Green fluorescence: cytoskeleton actin. Red fluorescence: TLR4 and NALP3. Blue fluorescence: cell nuclei. Scale bar: 20 µm.

**Figure 5:**
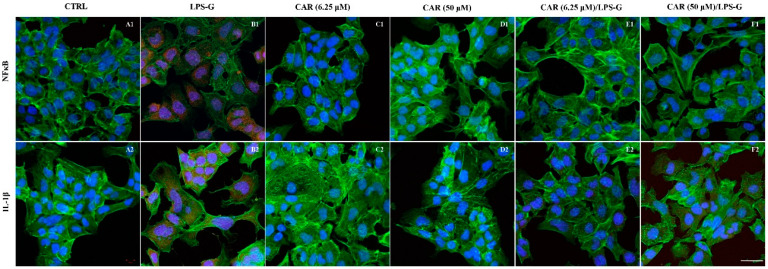
IL1-β/NFκB signaling pathway and differences in the protein levels in HL-1 cell lines. Expression of IL1-β and NFκB, analyzed by confocal microscopy (**A1**–**F1**). IL1-β expression in untreated cells (CTRL), HL-1 treated with LPS-G, CAR (6.25 μM), CAR (50 μM), CAR (6.25 μM)/LPS-G, CAR (50 μM)/LPS-G. (**A2**–**F2**) NFκB expression in untreated cells (CTRL), HL-1 treated with LPS-G, CAR (6.25 μM), CAR (50 μM), CAR (6.25 μM)/LPS-G, CAR (50 μM)/LPS-G. Green fluorescence: cytoskeleton actin. Red fluorescence: TLR4 and NALP3. Blue fluorescence: cell nuclei. Scale bar: 20 µm.

**Figure 6:**
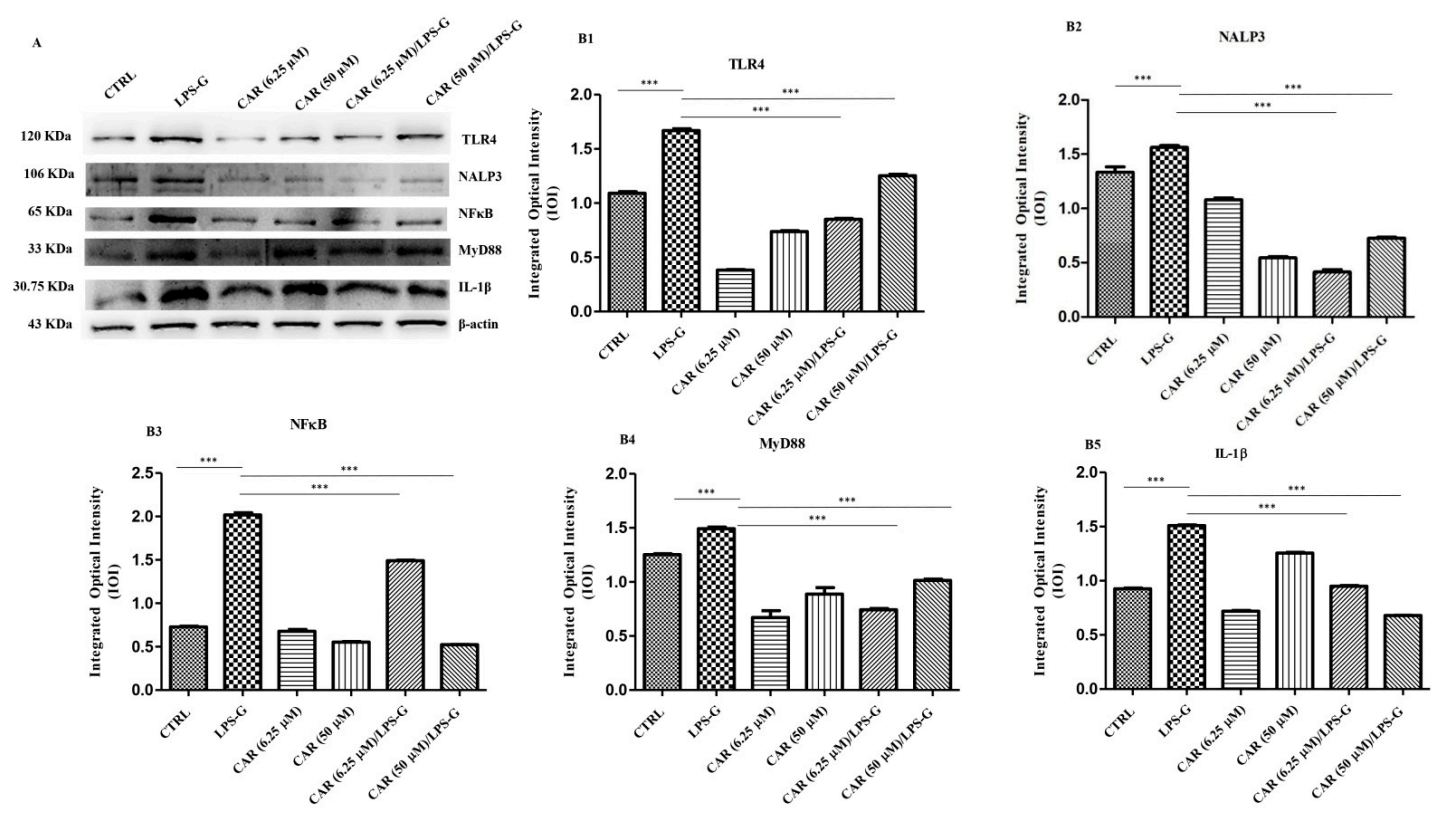
Western blotting analysis. (**A**) TLR4, NALP3, NFκB, MyD88, and IL1-β protein expression in the HL-1 cell line treated with LPS-G alone or in co-treatment with CAR. Each membrane was probed with β-actin antibody to verify the loading consistency. Western blot is representative of three different experiments. (**B1**–**B5**) Histograms represent densitometric measurements of proteins bands expressed as the integrated optical intensity (IOI) mean of three separate experiments. The error bars show the standard deviation (±SD). Densitometric values analyzed by ANOVA (post hoc application of Tukey’s multiple comparison test) returned significant differences. *** *p* < 0.001.

The immunofluorescence results showed that the TLR4/NFκB/NALP3/IL-1β pathway was expressed significantly in HL-1 treated with LPS-G for 24 h compared to the untreated cells. Cells treated with CAR alone at 6.25 and 50 µM or in co-treatment with CAR and LPS-G reported a severe decrease in proteins involved in the inflammation process (Figure 4 and Figure 5). These results were confirmed by Western blot analysis, where the HL-1 cell line treated with LPS-G showed higher expression of TLR4/NFκB/NALP3/IL-1β compared to the CTRL sample. On the contrary, CAR administration alone did not influence the protein levels similar to the untreated HL-1 cells (Figure 6).

### 3.3. CAR Reduces Reactive Oxygen Species Production in LPS-G-Treated HL-1 Cells

**Figure 7:**
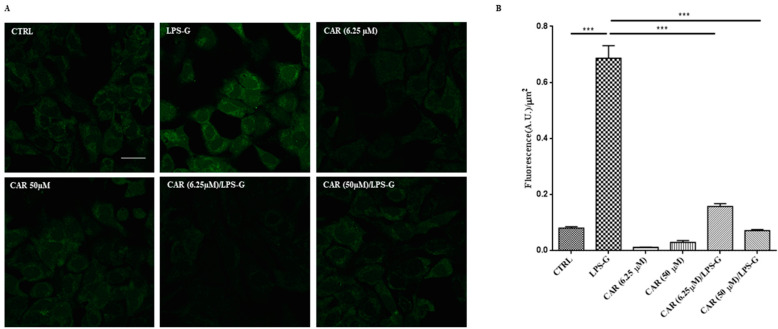
ROS measurements in HL-1 cells. (**A**) Representative images of H2DCFDA-loaded cells. Scale bar = 20 µm. (**B**) Quantitative analysis of ROS production calculated as the arbitrary unit of fluorescence per cell surface unit (Fluorescence A.U./µm^2^). Data are expressed as mean ± S.E.M (CTRL n = 195, LPS-G n = 237, CAR (6.25 µM) n = 792; CAR (50 µM) n = 286; CAR (6.25 µM)/LPS-G n = 188, CAR (50 µM)/LPS-G n = 252; *** *p* < 0.001).

The amount of ROS generation promoted by the treatment with LPS-G was assessed in the HL-1 cell line loaded with cell-permeable H2DCFDA. This fluorescent probe, once activated by intracellular esterases, became active to react with oxidative species. Here, the H2DCFDA is transformed to the extremely fluorescent 2′,7′-dichlorofluorescein (DCF) by ROS and the increase in fluorescence signals. Pictures were taken of live cells using confocal microscopy as evidenced in Figure 7A and the fluorescence signal was analyzed offline. The quantitative results (Figure 7B) show a notable increase in the ROS amount in HL-1 cells treated with 5 µg mL^−1^ LPS-G compared to the control condition. Interestingly, the co-incubation of LPS-G together with CAR at 6.25 and 50 µM counteracted the LPS-G increase in ROS production while CAR alone did not affect the production of ROS.

## 4. Discussion

It is well known that cardiovascular disease might be induced by long-term inflammation, with the latter being controlled by different molecules that promote inflammation, such as cytokines and angiotensin receptors.

Research on essential oils has attracted significant interest from the scientific community due to its potential regarding the identification of novel active molecules for the treatment of cardiovascular illnesses, such as myocardial infarction, arterial hypertension, angina pectoris, and heart breakdown. Different mechanisms have been identified regarding the role of essential oils and their main active components in supporting the health of the cardiovascular system [25].

In a paper published by Lee et al. (2006), it was evidenced that *Porphyromonas gingivalis* impairs cardiomyoblasts H9c2 through the promotion of hypertrophy factors and stimulation of the MMP9 fibrosis molecule. A previous work demonstrated that cardiomyopathy stimulated by *Porphyromonas gingivalis* in part includes p38 MAPK induction [26].

*Porphyromonas gingivalis* pathogenesis widely depends on its different virulence factors, comprising its specific structure, such as lipopolysaccharide, heat shock, fimbriae, and proteins, and secretory factors, such as gingipains and outer membrane vesicles [27]. The inflammatory response induced by LPS-G is a widespread in vitro model.

Numerous signaling pathways are implicated in different cellular processes that can eventually promote the release of inflammatory molecules and could instigate tissue damage, which can be stimulated by outer membrane endotoxins.

In our work, LPS-G stimulation was responsible for promoting an inflammatory response in an in vitro model of the HL-1 cell line, which activated the inflammatory TLR4/NALP3/NFκB/IL-1β pathway and ROS production. Based on the literature, LPS-G plays a key role in inducing inflammation and stimulating cells to secrete proinflammatory cytokines, such as IL-1β, TNF-α, and IL-6. LPSs are known to instigate proinflammatory cytokine formation, mainly through TLR4 and NFkB activation [28].

NALP3 is a member of NOD-like receptors (NLRs; set of intracellular PRRs), which, upon activation, assemble into a high-molecular-weight multi-protein caspase-1-activating platform known as the NALP3 inflammasome that orchestrates maturation of highly potent proinflammatory cytokines, such as IL-1β and IL-18 [29]. The NALP3 inflammasome is controlled by the existence of molecular patterns that are correlated with damage and starts or intensifies the inflammatory reaction throughout IL-1β and/or IL-18 formation.

Furthermore, NALP3, being the most fully characterized inflammasome, is implicated in intracellular inflammation; it can be stimulated by several bacterial ligands, including LPS-G [30]. Hence, unraveling the cellular and molecular aspects of NALP3 inflammasome activation and inhibition are crucial to the discovery and development of therapeutic candidates targeting NALP3 in different inflammatory disease conditions. The inflammatory reactions can be instigated by a sensor factor belonging to the NALP3 inflammasome. In tissue damage, the NALP3 inflammasome and TLR4 signaling have a crucial role [31]. TLR4 acts as a vital factor that stimulates the inflammatory reaction in the body. The suppression or downregulation of the NALP3/TLR4 signaling pathway represents a possible target in different long-term illnesses [32]. TLR4 stimulation instigates the myeloid differentiation factor 88 (MyD88) signaling pathway, which promotes rapid activation of NFκB, which consequently leads to an increase in IL-18, IL-6, IL-1β, tumor necrosis factor-α (TNF-α), and monocyte chemotactic protein-1 (MCP-1) [33]. ROS were proposed as the common signal for NALP3 inflammasome activation since most NALP3 stimuli can instigate ROS in treated cells, and lysosomal NADPH oxidase was primarily assumed to be the cause of ROS generation.

Our in vitro data suggest that HL-1 cells treated with LPS-G alone show increased ROS production and enhanced expression of TLR4/NALP3/NFκB/IL-1β. Instead, when the HL-1 cells were treated with CAR or in co-treatment with CAR/LPS-G, CAR being a natural antioxidant, the total ROS production in LPS-G-treated HL-1 cells was reduced and a reduction in the release of inflammatory mediators, such as TLR4/NALP3/NFκB/IL-1β, was shown as reported by confocal microscopy and Western blot analysis. These results obtained may suggest the possible pharmacological benefit of maintaining mitochondrial function with CAR, a natural antioxidant that attenuates NALP3 activation.

The present results suggest that CAR instigates anti-inflammatory outcomes in HL-1 cells through the generation of inflammatory factors that modulate the TLR4/NALP3/NFκB/IL-1β pathway and by decreasing ROS production. The current work aimed to investigate the possible therapeutic benefit of CAR in a cardiomyocyte cellular model, modulating the inflammatory effects induced by LPS-G through TLR4/NALP3/NFκB/IL-1β and ROS production. This biological outcome was, for the first time, investigated in HL-1 cells, whereas data for other terpenoids are not available yet. Conversely, CAR and some other structurally similar phenolic components of plant essential oils, such as thymol, eugenol, vanillin, and guaiacol, were tested on an L-type Ca^2+^ current in isolated ventricular cardiomyocytes in the concentration range of 10–1000 μM. Among these natural compounds, CAR was the most effective in the concentration-dependent and reversible suppression of cardiac Ca^2+^ currents, thus suggesting that this effect on cardiomyocytes is strongly related to the chemical functionalization of the core nucleus of terpenoids (CAR > thymol > eugenol) [34] (thymol (a regioisomer of CAR) also displayed concentration-dependent inhibitory effects on K^+^ currents in ventricular cardiomyocytes) [35]. Lastly, thymol and CAR were shown to act as inhibitors of protein and lipid oxidation induced by hydroxyl radicals to myofibrillar proteins; the mechanism was related to the reduction of lipid-derived volatile carbonyls and the formation of protein crosslinking [36].

## 5. Conclusions

CAR displayed a beneficial effect in an in vitro model on HL-1 cardiomyocytes treated with LPS-G. The protective role revealed by CAR may be attributed to its antioxidant and anti-inflammatory properties. Our work suggests that CAR could provide a significant beneficial effect by modulating the inflammatory effects promoted by LPS-G through activation of the TLR4/NALP3/NFkB/IL-1β signaling pathways and ROS production.

## Data Availability

Data is contained within the article or supplementary material.

## Supplementary Materials

The following supporting information can be downloaded at: https://www.mdpi.com/article/10.3390/antiox11020386/s1, Video S1: HL-1 cell culture.

## References

[1] Parnas M., Peters M., Dadon D., Lev S., Vertkin I., Slutsky I., Minke B. (2009). Carvacrol is a novel inhibitor of *Drosophila* TRPL and mammalian TRPM7 channels. Cell Calcium.

[2] Sisto F., Carradori S., Guglielmi P., Traversi C.B., Spano M., Sobolev A.P., Secci D., Di Marcantonio M.C., Haloci E., Grande R. (2020). Synthesis and Biological Evaluation of Carvacrol-Based Derivatives as Dual Inhibitors of *H. pylori* Strains and AGS Cell Proliferation. Pharmaceuticals.

[3] Sharifi-Rad M., Varoni E.M., Iriti M., Martorell M., Setzer W.N., Contreras M.D., Salehi B., Soltani-Nejad A., Rajabi S., Tajbakhsh M. (2018). Carvacrol and human health: A comprehensive review. Phytother. Res..

[4] Mouwakeh A., Telbisz A., Spengler G., Mohacsi-Farkas C., Kisko G. (2018). Antibacterial and Resistance Modifying Activities of *Nigella sativa* Essential Oil and its Active Compounds Against *Listeria monocytogenes*. In Vivo.

[5] Boskabady M.H., Alitaneh S., Alavinezhad A. (2014). *Carum copticum* L.: A Herbal Medicine with Various Pharmacological Effects. BioMed Res. Int..

[6] Marchese A., Arciola C.R., Coppo E., Barbieri R., Barreca D., Chebaibi S., Sobarzo-Sanchez E., Nabavi S.F., Nabavi S.M., Daglia M. (2018). The natural plant compound carvacrol as an antimicrobial and anti-biofilm agent: Mechanisms, synergies and bio-inspired anti-infective materials. Biofouling.

[7] Fratini F., Casella S., Leonardi M., Pisseri F., Ebani V.V., Pistelli L., Pistelli L. (2014). Antibacterial activity of essential oils, their blends and mixtures of their main constituents against some strains supporting livestock mastitis. Fitoterapia.

[8] Baser K.H.C. (2008). Biological and Pharmacological Activities of Carvacrol and Carvacrol Bearing Essential Oils. Curr. Pharm. Design..

[9] Hou N., Mai Y.P., Qiu X.X., Yuan W.C., Li Y.L., Luo C.F., Liu Y., Zhang G.P., Zhao G.J., Luo J.D. (2019). Carvacrol Attenuates Diabetic Cardiomyopathy by Modulating the PI3K/AKT/GLUT4 Pathway in Diabetic Mice. Front. Pharmacol..

[10] De Andrade T.U., Brasil G.A., Endringer D.C., da Nobrega F.R., de Sousa D.P. (2017). Cardiovascular Activity of the Chemical Constituents of Essential Oils. Molecules.

[11] Costa H.A., Dias C.J.M., Martins V.D., Araujo S.A., da Silva D.P., Mendes V.S., Oliveira M.N.S., Mostarda C.T., Borges A.C.R., Ribeiro R.M. (2021). Effect of treatment with carvacrol and aerobic training on cardiovascular function in spontaneously hypertensive rats. Exp. Physiol..

[12] Dias C.J., Costa H.A., Alves Dias-Filho C.A., Ferreira A.C., Rodrigues B., Irigoyen M.C., Romao Borges A.C., de Andadre Martins V., Branco Vidal F.C., Ribeiro R.M. (2022). Carvacrol reduces blood pressure, arterial responsiveness and increases expression of MAS receptors in spontaneously hypertensive rats. Eur. J. Pharmacol..

[13] Li Z.L., Hua C., Pan X.Q., Fu X.J., Wu W. (2016). Carvacrol Exerts Neuroprotective Effects Via Suppression of the Inflammatory Response in Middle Cerebral Artery Occlusion Rats. Inflammation.

[14] Canbek M., Uyanoglu M., Bayramoglu G., Senturk H., Erkasap N., Koken T., Uslu S., Demirustu C., Aral E., Baser K.H.C. (2008). Effects of carvacrol on defects of ischemia-reperfusion in the rat liver. Phytomed. Int. J. Phytother. Phytopharm..

[15] Chen Y.P., Ba L.N., Huang W., Liu Y., Pan H., Ming Yao E., Shi P.L., Wang Y., Li S.Z., Qi H.P. (2017). Role of carvacrol in cardioprotection against myocardial ischemia/reperfusion injury in rats through activation of MAPK/ERK and Akt/eNOS signaling pathways. Eur. J. Pharmacol..

[16] Mezzaroma E., Abbate A., Toldo S. (2021). NLRP3 Inflammasome Inhibitors in Cardiovascular Diseases. Molecules.

[17] Marconi G.D., Fonticoli L., Guarnieri S., Cavalcanti M.F.X.B., Franchi S., Gatta V., Trubiani O., Pizzicannella J., Diomede F. (2021). Ascorbic Acid: A New Player of Epigenetic Regulation in LPS-gingivalis Treated Human Periodontal Ligament Stem Cells. Oxid. Med. Cell. Longev..

[18] Chen T.S., Kuo C.H., Battsengel S., Pan L.F., Day C.H., Shen C.Y., Chung L.C., Padma V.V., Yao C.K., Lin Y.M. (2018). Adipose-derived stem cells decrease cardiomyocyte damage induced by *Porphyromonas gingivalis* endotoxin through suppressing hypertrophy, apoptosis, fibrosis, and MAPK markers. Environ. Toxicol..

[19] Zizzari V.L., Marconi G.D., De Colli M., Zara S., Zavan B., Salini V., Fontana A., Cataldi A., Piattelli A. (2015). In Vitro Behavior of Primary Human Osteoblasts Onto Microrough Titanium Surface. Implant. Dent..

[20] Trubiani O., Toniato E., Di Iorio D., Diomede F., Merciaro I., D’Arcangelo C., Caputi S. (2012). Morphological analysis and interleukin release in human gingival fibroblasts seeded on different denture base acrylic resins. Int. J. Immunopathol. Pharmacol..

[21] Sinjari B., Pizzicannella J., D’Aurora M., Zappacosta R., Gatta V., Fontana A., Trubiani O., Diomede F. (2019). Curcumin/Liposome Nanotechnology as Delivery Platform for Anti-inflammatory Activities via NFkB/ERK/pERK Pathway in Human Dental Pulp Treated with 2-HydroxyEthyl MethAcrylate (HEMA). Front. Physiol..

[22] Marconi G.D., Diomede F., Pizzicannella J., Fonticoli L., Merciaro I., Pierdomenico S.D., Mazzon E., Piattelli A., Trubiani O. (2020). Enhanced VEGF/VEGF-R and RUNX2 Expression in Human Periodontal Ligament Stem Cells Cultured on Sandblasted/Etched Titanium Disk. Front. Cell Dev. Biol..

[23] Marconi G.D., Gallorini M., Carradori S., Guglielmi P., Cataldi A., Zara S. (2019). The Up-Regulation of Oxidative Stress as a Potential Mechanism of Novel MAO-B Inhibitors for Glioblastoma Treatment. Molecules.

[24] Diomede F., Marconi G.D., Guarnieri S., D’Attilio M., Cavalcanti M., Mariggio M.A., Pizzicannella J., Trubiani O. (2019). A Novel Role of Ascorbic Acid in Anti-Inflammatory Pathway and ROS Generation in HEMA Treated Dental Pulp Stem Cells. Materials.

[25] Saljoughian S., Roohinejad S., Bekhit A.E.A., Greiner R., Omidizadeh A., Nikmaram N., Khaneghah A.M. (2018). The effects of food essential oils on cardiovascular diseases: A review. Crit. Rev. Food Sci..

[26] Chen T.S., Battsengel S., Kuo C.H., Pan L.F., Lin Y.M., Yao C.H., Chen Y.S., Lin F.H., Kuo W.W., Huang C.Y. (2018). Stem cells rescue cardiomyopathy induced by P-gingivalis-LPS via miR-181b. J. Cell Physiol..

[27] Mei F., Xie M.R., Huang X.F., Long Y.L., Lu X.F., Wang X.L., Chen L.L. (2020). *Porphyromonas gingivalis* and Its Systemic Impact: Current Status. Pathogens.

[28] Yu G.M., Kubota H., Okita M., Maeda T. (2017). The anti-inflammatory and antioxidant effects of melatonin on LPS-stimulated bovine mammary epithelial cells. PLoS ONE.

[29] Kelley N., Jeltema D., Duan Y.H., He Y. (2019). The NLRP3 Inflammasome: An Overview of Mechanisms of Activation and Regulation. Int. J. Mol. Sci..

[30] Diomede F., Thangavelu S.R., Merciaro I., D’Orazio M., Bramanti P., Mazzon E., Trubiani O. (2017). *Porphyromonas gingivalis* lipopolysaccharide stimulation in human periodontal ligament stem cells: Role of epigenetic modifications to the inflammation. Eur. J. Histochem..

[31] Soundara Rajan T., Giacoppo S., Diomede F., Bramanti P., Trubiani O., Mazzon E. (2017). Human periodontal ligament stem cells secretome from multiple sclerosis patients suppresses NALP3 inflammasome activation in experimental autoimmune encephalomyelitis. Int. J. Immunopathol. Pharmacol..

[32] Yang J., Wise L., Fukuchi K.I. (2020). TLR4 Cross-Talk With NLRP3 Inflammasome and Complement Signaling Pathways in Alzheimer’s Disease. Front. Immunol..

[33] Kawai T., Takeuchi O., Fujita T., Inoue J., Muhlradt P.F., Sato S., Hoshino K., Akira S. (2001). Lipopolysaccharide stimulates the MyD88-independent pathway and results in activation of IFN-regulatory factor 3 and the expression of a subset of lipopolysaccharide-inducible genes. J. Immunol..

[34] Magyar J., Szentandrassy N., Banyasz T., Fulop L., Varro A., Nanasi P.P. (2004). Effects of terpenoid phenol derivatives on calcium current in canine and human ventricular cardiomyocytes. Eur. J. Pharmacol..

[35] Magyar J., Szentandrassy N., Banyasz T., Fulop L., Varro A., Nanasi P.P. (2002). Effects of thymol on calcium and potassium currents in canine and human ventricular cardiomyocytes. Br. J. Pharmacol..

[36] Lahmar A., Akcan T., Chekir-Ghedira L., Estevez M. (2018). Molecular interactions and redox effects of carvacrol and thymol on myofibrillar proteins using a non-destructive and solvent-free methodological approach. Food Res. Int..

